# A Novel Approach to Female Genital Mutilation Reconstruction with Fat Grafting and Adipose Stem Cell Therapies: A Minimally Invasive Solution with a Potential Impact on Millions of Women Worldwide

**DOI:** 10.1007/s00266-025-04895-9

**Published:** 2025-05-23

**Authors:** Aurora Almadori, Esther Hansen, Peter Butler, Marzia Salgarello

**Affiliations:** 1 Division of Surgery & Interventional Science, Centre for Nanotechnology and Regenerative Medicine, University College London, London, UK; 2 Department of Plastic Surgery, Royal Free NHS Foundation Trust Hospital, London, UK; 3 Charles Wolfson Centre for Reconstructive Surgery, Royal Free Hospital, London, UK; 4 Plastic Surgery Unit, Department of Women and Child Health, Fondazione Policlinico Universitario A. Gemelli IRCCS, Rome, Italy; 5 Clinical Psychology, Department of Plastic Surgery, Royal Free NHS Foundation Trust Hospital, London, UK

**Keywords:** Female genital mutilation, Vulva, Scar, Fat grafting, Adipose-derived stem cells (ASCs), Stromal vascular fraction (SVF)

## Abstract

**Background:**

FGM is an issue of increasing concern also in countries where it is not traditionally practiced. Vulvar scarring is the most common long-term effect associated with FGM, representing one of the main unmet issues in FGM women’s health. Regenerative therapies based on the use of adipose-derived stem cells are considered the standard of care for ameliorating scarring and fibrosis. This study aimed to explore the potential of fat grafting in the treatment of post-FGM vulvar scars.

**Methods:**

Thirteen FGM survivors with vulvar scars underwent autologous fat grafting and were assessed using the Vulvar architecture severity scale (VASS), Female genital self-image scale (FGSIS), Female sexual function index (FSFI), and Hospital anxiety and depression scale (HADS).

**Results:**

At an average follow-up of 12.23 months (± 3.03), clinical results (VASS) showed a significant improvement in all vulvar aesthetic units treated with FG (*p *< 0.001). Patients reported improvements in genital-related self-image (FGSIS) (*p *= 0.001), sexual function (FSFI) (*p *= 0.019), and psychological well-being (HADS) (*p *= 0.002).

**Conclusions:**

Fat grafting ameliorates FGM-related vulvar scars and improves volumetric contouring of vulvar aesthetic units, with a positive effect on women’s quality of life. This minimally invasive intervention has far-reaching implications, providing a cost-effective solution accessible even in low-resource settings to potentially improve the overall well-being of millions of women living worldwide with a form of FGM. The results of this study warrant further testing in future clinical trials.

**Level of Evidence IV:**

This journal requires that authors assign a level of evidence to each article. For a full description of these Evidence-Based Medicine ratings, please refer to the Table of Contents or the online Instructions to Authors www.springer.com/00266.

## Introduction

### FGM and Vulvar Scarring

**Fig. 1:**
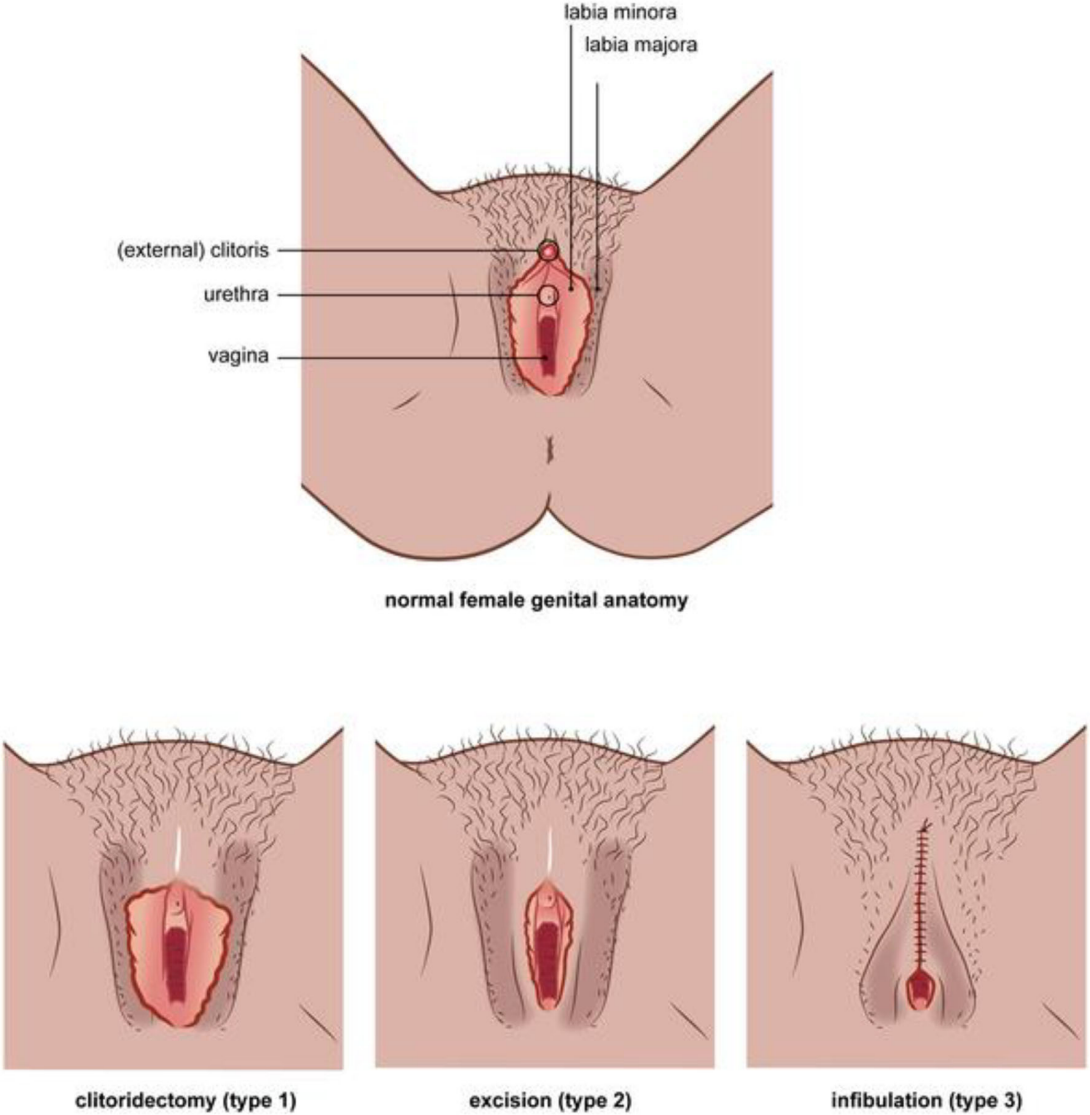
FGM classification. Type I-involves the removal of the clitoral hood or the clitoral glans, a procedure also known as clitoridectomy, even when it pertains solely to the external portion of the clitoris. Type II-encompasses the total or partial excision of the labia minora, which may occur with or without the removal of the clitoral area, commonly referred to as excision. Type III-consists of narrowing the vaginal opening through cutting and suturing the inner and/or outer labia, which may or may not include the ablation of the clitoral area, as well as the potential excision of the inner labia; this is often termed infibulation. Type IV-includes any other harmful practices directed at the female genitalia for non-medical reasons, such as pricking, piercing, cauterization, or incision of the clitoris or labia, scraping of the tissue around the vaginal opening or cutting into the vaginal wall, and the insertion of corrosive substances or herbs into the vagina aimed at inducing bleeding, tightening, or narrowing. Reproduced from Kawous et al 2020 [2]

Female Genital Mutilation (FGM) is defined as any treatment that involves the removal of a woman’s external genitalia for cultural or other non-medical reasons [1]. The World Health Organization (WHO) classifies FGM into four types (Fig. 1). It is estimated that 135 million women worldwide are subjected to some form of FGM, and with COVID-19 pandemic cases have increased owing to school closures, movement restrictions, and lockdowns that were seen as opportunities to carry out FGM undetected [2].

In the past, FGM was considered a phenomenon mainly linked to Africa, the Middle East (Saudi Arabia, Iraq, Yemen, Oman,), and Asia (Malaysia, Indonesia, and India). Because of migration flows, FGM is now widespread in areas where FGM is not traditionally practiced, with an estimated prevalence of 513,000 women in the USA [3], 137,000 in the UK [4], and 87,000 in Italy [5]. The economic cost for public health systems can be significant, with recent reports showing that one in three women who experience the medical consequences of FGM requires treatment and hospitalization [6].

**Fig. 2:**
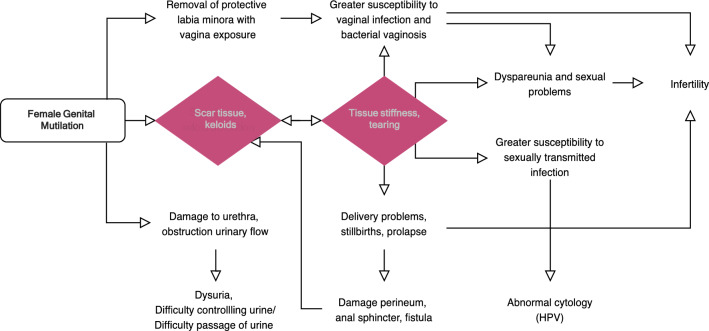
Effects of vulvar scarring on the health and well-being of FGM survivors. This framework demonstrates the potential effects of vulvar scar on FGM women’s health. The presence of scar tissue plays a crucial role in overall morbidity, primarily due to the increased stiffness of the fibrotic scar tissues, positioning it as a key therapeutic target. Adapted and reproduced with permission from [13]

FGM is typically carried out on girls aged 4 and 13 by traditional women who lack anatomical expertize, use rudimental tools, perform the procedure in non-sterile settings, and do not administer anesthetic. It is often a traumatic experience that significantly impairs women’s quality of life. The majority of FGM survivors presents acute and long-term sequelae. One of the main long-term consequences of FGM is hypertrophic scar formation in response to tissue damage, which is aggravated by the use of tools without a clear cut, and the unsterile setting, both affecting negatively wound healing [6]. A recent meta-analysis showed that FGM survivors have a significantly greater risk of scar formation than those without FGM [7]. Hypertrophic scarring’s prevalence has been reported being 27.5% with FGM type II, 16.9% with FGM type III, and 15.1% in women with FGM type I [6–8]. Keloids, on the other hand, are more common in FGM type III than in types I or II [7, 9–11]. In the vulvar and perineal area, tissue stiffness and retraction of the scarred skin and surrounding tissues might be detrimental to FGM survivors. The impaired tissue elasticity is not only associated with chronic symptoms such as pain, itching, and dyspareunia, but it can also have an impact on the physiologic functionalities with obstruction to urinary and menstrual flow, and a higher complication rate during delivery potentially leading to additional tearing and perineal damage (Fig. 2) [12, 13]. While multiple reconstructive approaches have been described to reinstate the removed anatomical vulvar structures (clitoral or inner labia reconstruction), the management of vulvar scars post-FGM has not been properly addressed so far. In this context, addressing vulvar scarring represents one of the main unmet needs in FGM women’s health and a crucial therapeutic target.

### Scar Tissue Regeneration with Adipose Stem Cell-Based Therapies

Regenerative therapies based on processed adipose tissue have been used in multiple types of scars and fibrotic conditions for tissue regeneration and repair [14–20]. Adipose tissue is abundant with Adipose-derived stem cells (ASCs), a subset of multipotent mesenchymal stem cells [21]. According to in vitro and clinical research, ASCs can ameliorate dermal fibrosis, promote wound healing, and repair tissue damage [22]. Owing to their proangiogenic, anti-inflammatory, and immunomodulatory effects, ASCs are now considered an optimal treatment for the regeneration of scarred tissues [23].

**Fig. 3:**
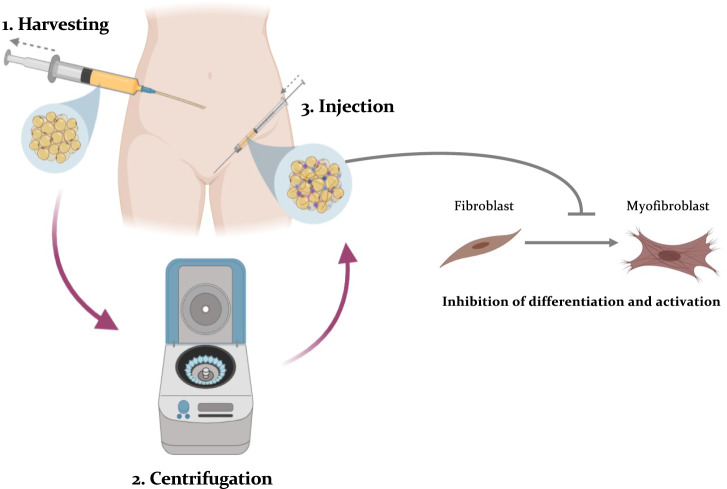
Vulvar fat grafting technique. The procedure is divided into three stages. The initial stage involves the removal of fat from a donor site, typically the abdomen or thighs, using a liposuction cannula (1). The second stage focuses on processing the harvested lipoaspirate through centrifugation. The aim of centrifugation is to allow the separation of the parts to be discarded (oil and cellular debris) from the part to be used for injection that is rich with ASCs, pericytes and stromal cells (2). The selected fraction is then injected in the vulvar tissues (3). Different techniques are available to process the adipose tissue, such as centrifugation, washing, filtration, decantation, and fractionation. In this series of patients, macro- and micro-FG were adopted and obtained with centrifugation. The injected lipoaspirate has a volumetric effect but also a regenerative effect. The latter is mediated by the ASCs that exert a paracrine effect on the local fibroblasts, downregulating their activation into myofibroblasts and remodeling the scar

While treatment with ASCs requires cells expansion through culture and collagenase digestion, fat grafting (FG) is more readily available, requires less tissue manipulation and as such is compliant with regulatory bodies, does not require complex lab facilities, and is less costly. It involves using liposuction to remove adipose tissue from specific body parts, followed by the injection of the fat fraction containing adipose stem cells into the scarred recipient area (Fig. 3). The advantages of this technique, besides its proven efficacy, are that it is minimally invasive, is performed as day case, is well tolerated by patients, presents low morbidity, with contained costs. For these reasons, FG is considered a well-established technique routinely adopted as gold standard technique to improve dermal fibrosis in hypertrophic scars, radiation-induced fibrosis, burns, traumatic injuries, scleroderma, lichen sclerosus, graft versus host disease, and Dupuytren’s contracture [14, 24–28]. The rationale behind implementing FG in FGM relies mainly in its regenerative properties to improve the scarred skin, but also to enhance volumetric defects caused by scar retraction or vulvar structures removal.

The purpose of this pilot study was to assess the safety and efficacy of FG in FGM survivors who exhibit vulvar scarring following FGM.

## Materials and Methods

This study was approved by our institutional ethics committee (references P/906/CE/2012 and 1403805) and performed in accordance with the ethical standards of the 1964 Declaration of Helsinki and its later amendments.

### Objectives

The overall objective of this pilot study was to preliminarily explore the effect of FG in FGM vulvar scars to pave the way for a subsequent clinical trial. The primary outcome consisted in assessing the efficacy of FG in the treatment of vulvar scarring due to FGM. This was measured with validated patient-reported outcome measures (PROMs) and validated physician-based assessments. The secondary outcome was the assessment of the safety of FG in FGM, defined as a complication of the procedure.

### Inclusion and Exclusion Criteria

All FGM survivors presenting with vulvar scars were considered eligible, irrespective of the type of FGM they underwent. In our department, FG for vulvar scarring is performed in women between 18 and 70 years of age, not pregnant, and without any active infection or cancer disease.

### Outcome Assessment

Women who underwent FG were assessed using multiple PROMs to evaluate genital self-image, sexual function, and psychological status. The FGSIS-Female genital self-image scale is a tool for measuring women’s self-perception of genital appearance [29]. The scale consists of seven items rated on a 4-point scale. The FSFI-Female sexual function index is a scale assessing sexual feelings and responses [30]. It is composed of multiple subdomains: desire, arousal, lubrication, orgasm, satisfaction, and pain [30]. Hospital anxiety and depression scale (HADS) evaluates anxiety and depression [31]. It is composed of 14 items, seven related to anxiety and seven to depression [31]. To systematically assess the extent of FGM, the Vulvar architecture severity scale (VASS) was adopted. This physician-based scale has been previously developed and validated by our team to comprehensively assess the vulvar area in lichen sclerosus with an aesthetic unit approach, given that each anatomical unit is assessed independently [32]. The VASS assessment took place before surgery and at the follow-up appointment at least six months after FG, either through direct patient examination or by reviewing their photographs.

### Surgical Technique

**Fig. 4:**
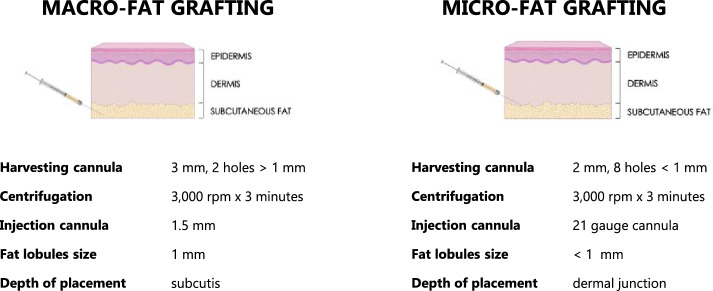
Characteristics of the adopted surgical techniques. In this study macro-FG and the micro-FG were used. The primary distinction lies in the dimensions of the fat lobules, which can either be larger or smaller, allowing for the placement occurring at a deeper or more superficial level

In this series, the surgical techniques utilized were macro- and micro-FG (Figs. 3, 4). The difference between macro- and micro-FG consists on the size of the fat lobules obtained (Fig. 4). A cannula 3 mm in diameter, with two 1 mm holes (Blink) was used to obtain macro-fat, and, after centrifugation, the lipoaspirate was injected subcutaneously using a 1.5 mm cannula [33]. Micro-fat was harvested using a cannula of 2 mm diameter with eight holes measuring less than 1 mm. After centrifugation, the lipoaspirate was then grafted more superficially into the dermal junction or directly within the scarred tissue with a 21-gauge cannula (St’rim) [14, 34]. Patients underwent the procedure under sedation or general anesthesia and were discharged on the same day.

### Statistical Analysis

Pre-treatment and post-treatment values were statistically analyzed using a paired t-test (nonparametric Wilcoxon matched-pairs signed rank test), considering *p*-values 0 < 0.05 as significant (Prism6 Software). The test was two-tailed at 95% confidence interval.

## Results

### Patients Details

Thirteen FGM survivors treated with FG completed the pre- and postoperative assessment. The mean patients’ age was 32.46 (± 6.39) years. Women presented with FGM types I (15.4%), II (38.5%), and III (46.1%). The details are presented in Table 1.

**Table 1:** Characteristics of included patients

No of patients	13
Age	Mean 32.46, SD ±6.39 Median 32, Range 21-45
No of treatment	Mean 1.38, SD ±0.65 Median 1, Range 1-2
Follow-up (months)	Mean 12.23, SD ±3.03 Median 12, Range 6-18
No of FGM type I	2 (15.4%)
No of FGM type II	5 (38.5%)
No of FGM type III	6 (46.1%)

### Reasons for Seeking and Undergoing Regenerative Surgery

FGM survivors were treated with autologous FG for multiple scenarios. The main complaints reported were related to symptoms such as itching, pain, dyspareunia, ‘feeling tension and retraction,’ oversensitivity of the neo-created clitoral glans (after clitoral reconstruction); and/or concerns regarding appearance including asymmetry, scar retraction, and feeling of having the vaginal opening too exposed after defibulation (Table 2). The indications for surgery were regeneration of retracting scar tissue, volumetric enhancement, asymmetry correction, and increase in the subcutaneous tissue bulk to allow subsequent reconstruction (Table 2).

**Table 2:** Indications for fat grafting and surgical details

Patient code	FGM type	Motivation for requesting FAT grafting	Indication for undergoing FAT grafting	Amount injected (ML)				
				Outer Labia	Inner Labia	Clitoral area	Posterior fourchette	Total amount
1	2	Symptoms (itching and dyspareunia); Appearance	Inner labia scar; Feeling of exposed vagina after defibulation	6	4	2	2	14
2	1	Symptoms (itching)	Clitoral hood scar; Volumetric enhancement	0	2	1,5		3,5
3	2	Symptoms (pain)	Inner/outer labia scar	4	2	1,5	1,5	9
4	3	Appearance	Inner/outer labia scar; Feeling of exposed vagina after defibulation	4	3	2	2	11
5	3	Symptoms (dyspareunia and ‘feeling of tension’); Appearance	Inner labia scar; Feeling of exposed vagina after defibulation	5	2	1	2	10
6	1	Symptoms (itching)	Clitoral hood scar	0	0	1	2,5	3,5
7	3	Symptoms (itching, pain and dyspareunia); Appearance	Post-defibulation scar in inner/outer labia and anterior fourchette	7	2	3	2	14
8	2	Appearance	Inner and outer labia scar	6	1	0	1	8
9	2	Appearance	Increase volumes inner labia remnants Correction of inner labia asymmetry	4	1,5	2	2	9,5
10	3	Symptoms (itching and dyspareunia); Appearance	Post-defibulation scar in inner labia; Feeling of exposed vagina after defibulation	7,5	1	1	0	9,5
11	2	Symptoms (pain and dyspareunia); Appearance	Increase volumes inner labia remnants; Post-defibulation scar anterior fourchette	8	2	1	0	11
12	3	Symptoms (pain and oversensitivity of the neo-created clitoral glans); Appearance	Tissue preloading (after clitoral reconstruction and before hood reconstruction); Inner labia scar	6	4	1	1,5	12,5
13	3	Symptoms (itching, pain and dyspareunia); Appearance	Inner labia and perineal scar; Feeling of exposed vagina after defibulation	8	1	1	2	12

### Surgical Details

Overall, the average amount injected was 9.8 ml (± 3.3), with a median of 10 ml (range 3.5–14). The subunit breakdown was as follows: outer labia, average 5.9 ml (± 1.5), median 6 (range 0–8); inner labia, average 2.1 ml (± 1), median 2 (range 0–4); clitoral area, average 1.5 ml (± 0.6), median 1.2 (range0–2); posterior fourchette, average 1.8 ml (± 0.4), median 2 (range 0–2.5) (Table 2).

### Outcome

Follow-up was on average 12.23 months (± 3.03), with a median of 12 (range 6–18) (Table 2). Overall, women exhibited with clinical improvement following treatment at varying degrees. The details of PROMS scores are illustrated in Table 4. Genital-related self-image resulted in a statistically significant improvement after FG (*p *= 0.001), particularly in the following items: satisfaction with appearance (*p *= 0.001); overall feeling about genitals (*p *= 0.001); and comfortability with showing genitals to a partner (*p *= 0.001). Similarly, women indicated a significant enhancement in their sexual functioning. (*p *= 0.019). The main subdomains reported as improved were orgasm (*p *= 0.012), pain (*p *= 0.012), and arousal (*p *= 0.039) (Table 4). Psychological status also improved in both anxiety (*p *= 0.001) and depression (*p *= 0.002) (Table 3).

**Table 3:** Patients reported outcome measures

QUESTIONNAIRE	Sub-domain	Before FG score (mean, ±SD)	After FG score (Mean, ±SD)	Score change (mean, ±SD)	*P* value
FGSIS	Total score	15 ±2.1	22.4 ±1.6	7.4 ±1.6	0.001 ***
	Overall feeling about genitals	1.8 ±0.6	3.3 ±0.6	1.5 ±0.8	0.001 ***
	Satisfaction with appearance	1.8 ±0.7	3.5 ±0.5	1.7 ±0.9	0.001 ***
	Comfortability with showing genitals to a partner	1.7 ±0.6	3.2 ±0.6	1.5 ±0.9	0.001 ***
	Smell	2.9 ±0.7	2.8 ±0.8	0.1 ±0.8	0.721 NS
	Functionality	2.1 ±0.6	3.2 ±0.6	1.1 ±0.6	0.001 ***
	Comfortability with clinical examination	2.9 ±0.8	3 ±0.7	0.1 ±0.6	0.673 NS
	Embarrassment about genitals	1.9 ±0.5	3.4 ±0.5	1.5 ±0.7	0.001 ***
FSFI	Total score	20.4 ±9.4	27.9 ±4.3	7.4 ±6.3	0.019 **
	Desire	3.2 ±1.7	3.9 ±1.2	0.7 ±0.7	0.041 *
	Arousal	3 ±2	4.4 ±1	1.4 ±1.2	0.039 *
	Lubrication	3.9 ±1.9	4.9 ±0.7	1 ±1.4	0.012 *
	Orgasm	3.3 ±1.7	4.8 ±1	1.4 ±1.5	0.012 *
	Satisfaction	3.8 ±1.5	5.4 ±0.5	1.6 ±1.6	0.099 NS
	Pain	3.3 ±1.8	4.6 ±0.9	1.3 ±1.5	0.012 *
HADS	Anxiety	10 ±4.3	7.5 ±3.2	2.5 ±2	0.001 ***
	Depression	9.5 ±4.3	6.8 ±2.9	2.6 ±2.3	0.002 **

The physician-based assessment reported an overall improvement in all vulvar aesthetic units treated with FG (*p *< 0.001) (Table 4). The most noticeable improvements were reported in the inner (*p *= 0.00002) and outer labia (*p *= 0.00009), followed by posterior fourchette (*p *= 0.002) and clitoral area (*p *= 0.003) (Table 4).

**Table 4:** Doctor reported outcome—vulvar architecture severity scale

VASS	Aesthetic unit	Before FG score Median (Range) Mean ±SD	After FG score median (Range) Mean ±SD	Score change median (Range) Mean ±SD	*p* Value
	Overall	8 (1-12) 7.09 ±3.09	3 (0-7) 2.77 ±1.88	5 (1-7) 4.31 ±1.8	0.00001 ***
	Clitoral area	2 (0-3) 1.85 ±0.99	1 (0-3) 1.31 ±1.32	1 (0-2) 0.54 ±0.52	0.003 **
	Outer labia	2 (0-3) 2 ±1.15	0 (0-1) 0.62 ±0.87	1 (0-3) 1.38 ±0.87	0.00009 ***
	Inner labia	2 (0-3) 1.92 ±1.04	0 (0-2) 0.46 ±0.66	2 (0-3) 1.46 ±0.78	0.00002***
	Posterior fourchette	1 (0-3) 1.31 ±1.03	0 (0-1) 0.38 ±0.51	1 (0-3) 0.92 ±0.86	0.002 **

### Complications

Overall, the procedure was well tolerated and no complications occurred. One patient (7.6%) presented with bruising at the donor site 14 days after the procedure, which resolved spontaneously without requiring any intervention or medication.

## Discussion

FGM is a complex and deeply rooted practice, with significant physical, psychological, and social consequences for affected women. The associated healthcare financial burden is substantial, with the WHO estimating that the annual cost of managing FGM-related complications reaches $1.4 billion globally [35]. The health complications vary depending on the type and severity of FGM, with vulvar scarring being a common issue that can significantly affect care costs. Although surgical interventions such as clitoral reconstruction, and labial reconstruction offer the potential for functional and aesthetic enhancement, several challenges remain [36]. Firstly, these techniques, particularly inner labia reconstruction, require specialized plastic surgery or microsurgical expertize, which is not always available in resource-limited settings. Additionally, these procedures often result in prolonged operative times and greater invasiveness, which may not be ideal for patients with previous trauma, and the higher costs associated with these techniques make them less sustainable particularly in developing countries. In contrast, fat grafting offers a less invasive, cost-effective solution with fewer complications, making it a preferred technique in FGM reconstruction, especially where resources and expertize are constrained. The minimally invasive nature of FG makes is a first-line treatment for FGM defects, aligning with the principle of the *‘reconstructive ladder’*, a fundamental concept in reconstructive surgery [44]. It emphasizes starting with the simplest and least invasive options, progressively moving to more complex surgical interventions based on the severity of the condition. This framework guides clinicians in choosing the most appropriate treatment approach for each patient. Moreover, inner labia reconstruction and clitoral reconstruction aim to reinstate the anatomy but overlook the role of scar tissue in determining surgical outcomes. In our experience the scar tissue-related problems are not directly proportional to the severity of FGM, with multiple women presenting with FGM type III (the most severe form) without severe issues related to the scar, and women with FGM type Ia (the mildest form) presenting with symptomatic scarring. Therefore, there is merit in addressing vulvar scar as a separate entity in the context of FGM survivors’ care. For example, complications arising during childbirth are more frequently associated with the presence of scar tissue and its reduced elasticity, rather than the mutilation itself (Fig. 2); indeed, FGM women have a higher risk of tearing compared to non-FGM women even when they undergo antenatal defibulation to open the vaginal passage. Scar tissue can also compromise the outcome of reconstructive surgery due to the impaired wound healing capacity of fibrotic tissues, leading to a higher incidence of wound dehiscence and other complications [37]. Therefore, scar tissue can be considered as one of the main unmet issues in FGM women’s health and addressing it represents a crucial therapeutic target in the care of FGM survivors.

**Fig. 5:**
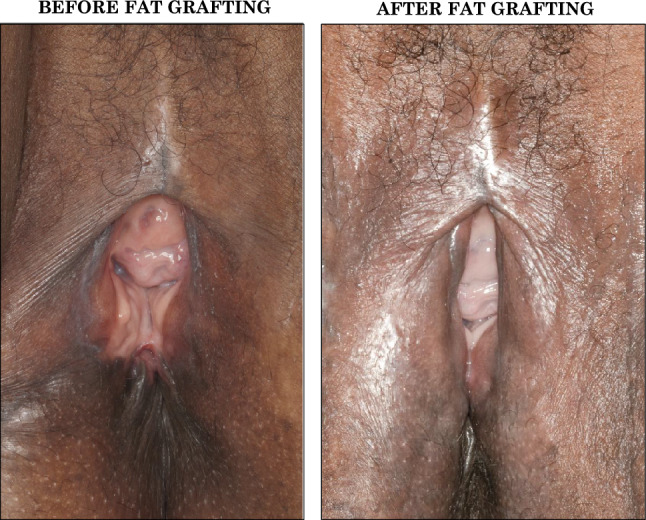
Representative clinical case 1: volumetric enhancement**.** The patient underwent inner labia resection, and suture of the outer labia including the clitoral hood. According to WHO classification this is Type 3; according to VASS the form is SEVERE in the clitoral area and inner labia (complete loss of the anatomical structures); MODERATE in the outer labia (presence of scar tissue + partial loss of anatomical structure). During pregnancy, she underwent antenatal defibulation and experienced traumatic childbirth. After delivery, she requested re-infibulation complaining a feeling of overly exposed vagina, along with the presence of scar tissue at the level of the inner and outer labia (left). After two fat grafting procedures, the outer labia volumes resulted increased with better covering of the vaginal opening, and the scar tissue was ameliorated (right). The woman reported improvement in genital self-image

**Fig. 6:**
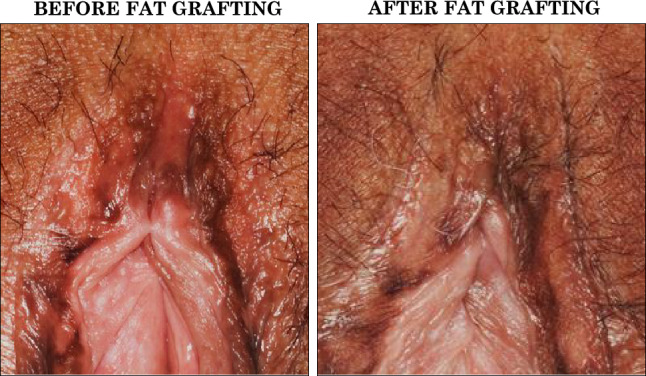
Representative clinical case 2: regenerative effect in scarred fibrotic tissue. The patient underwent partial clitoral hood resection. According to WHO classification this is Type 1A; according to VASS the form is MODERATE in the clitoral area (presence of scar tissue + partial loss of anatomical structure, in this case partial lack of clitoral hood). She presented with scar tissue in the clitoral area, adhesions in the clitoral hood, and lack of subcutaneous tissue bulk in the clitoral hood (left). After one treatment with fat grafting, the clitoral hood’s soft tissue bulk results increased, and the shape is reinstated. In addition to that, the tissue fibrosis is ameliorated, with a resolution of the skin adhesion (right). The woman reported an enhancement in both her itching symptoms and sexual functioning

ASCs have been proved to have a significant regenerative capacity for ameliorating fibrotic skin, including FGM-related vulvar scarring. Various surgical approaches have been explored to process the adipose tissue with the purpose of influencing the number of ASCs and fat viscosity, such as washing, filtering, decanting, or centrifugating. Although there is no evidence supporting the methods mentioned above, we chose to adopt centrifugation as the processing technique. This decision was based on our intention to follow the standardized method described by Coleman decades ago, as centrifuged lipoaspirate has been thoroughly characterized. These techniques offer flexibility in maximizing the augmentation or regeneration potential based on the treatment area and the desired outcome. In this study we adopted the standard macro-FG technique with larger fat lobules deeply placed to address volumetric defects (Fig. 4a), along with micro-FG with smaller fat lobules and ASCs to target areas with scarring, allowing for more superficial delivery (Fig. 4b). The adaptability of these methods ensures a highly personalizable approach, allowing for precise treatment of individual patient needs making it an effective treatment for all types of FGM. Its ability to simultaneously address multiple vulvar aesthetic units presents a unique advantage in FGM reconstruction. Based on the type of FGM, issue of concern, and desired outcome, FG can enhance both the functional and aesthetic aspects of the vulvar region by enhancing tissue elasticity and aiding in the overall appearance of the vulva (Table 5, Fig. 5). For example, in FGM Type III, where defibulation (reopening of the vaginal passage) is commonly performed, FG can complement this procedure not only by improving the quality of scar tissue but also by enhancing the overall vulvar shape. This is achieved by augmenting the outer labia, which reduces the sensation of an overly exposed vaginal opening after defibulation. Therefore, by restoring volume and improving skin quality, FG can help recreate a more natural vulvar appearance and reduce the physical and psychological discomfort often experienced by patients after defibulation (Fig. 6). This is particularly relevant for women from communities where a normal vaginal opening is not well perceived or is perceived with embarrassment. For this reason, women with FGM III after childbirth often request re-infibulation, which is illegal in most western countries [38, 39]. Offering labia minora/majora augmentation represents a minimally invasive way to address their appearance concerns, without compromising the vulvar functionality and mostly in compliance with our duty of care as health care providers.

**Table 5:** Versatility of fat grafting in FGM-related vulvar scar

Application	Rationale	Considerations	Type of FGM	Aesthetic sub-units that can be targeted
1. FGM-related scar	Regeneration of scar tissue: -ASCs’ effect on fibrotic fibroblasts; -Lipoaspirate’s effect of subcutaneous tissue bulk enhancement	FGM is often performed with rudimental tools, not sharp and non-sterile. These affect the wound healing and the subsequent scar formation. FGM is also performed by non-medically trained individuals, causing damage and scar also to adjacent structures.	I II III	Clitoral hood Inner labia Outer labia Posterior fourchette Perineum
2. Post-defibulation scar	Regeneration of scar tissue: ASCs’ effect on fibrotic fibroblasts; Lipoaspirate’s effect increase of subcutaneous tissue bulk	While defibulation procedure presents clear advantages regaining access to the vaginal opening, allowing normal urine and menstrual flow, and facilitating sexual intercourse, it can often leave women with additional vulvar scar.	II III	Inner labia Outer labia Posterior fourchette Perineum
3. Volumetric enhancement (functional and aesthetic)	Volumetric enhancement of the subcutaneous soft tissue bulk and projection of the treated areas	Particularly relevant after defibulation in women requesting a post-partum re-infibulation, complaining a feeling of ‘exposed vaginal opening’. The HCPs should to explain women that infibulation is not allowed in most countries, even if a woman has had it before and she asks for it as an adult capable of giving informed consent. Outer labia FG can increase their volume and facilitate the covering of the vaginal opening.	I II III	Clitoral hood Inner labia Outer labia Posterior fourchette Perineum
4. Tissue preloading with fat (preparation for subsequent multi-stage reconstruction)	Bonification of scarred fibrotic tissues with FG as preparation for further procedures, with two advantages: Improving tissue elasticity, hence reducing the risk of wound dehiscence (poor healing capacity of fibrotic tissues); Increase of subcutaneous tissue, allowing a subsequent adjacent tissue mobilization (i.e., local flap).	Clitoral reconstruction often does not include clitoral hood reconstruction. Women with a newly created clitoral glans often experience oversensitivity issues. A clitoral hood can be created with an advancement flap. Similarly, after FGM or defibulation women often complain a feeling of exposed vagina and request inner labia reconstruction. This can be performed with local flaps. However, if scar is present, the reduced tissue elasticity and the poor healing capacity of fibrotic scarred tissue make the reconstruction challenging. FG can be performed to increase the soft tissue bulk in the clitoral area/inner labia remnants or in the outer labia to improve the tissue quality and increase the subcutaneous tissue bulk, in order to allow further subsequent clitoral hood and/or inner labia reconstruction.	I II III	Clitoral hood Inner labia Outer labia Posterior fourchette Perineum

**Fig. 7:**
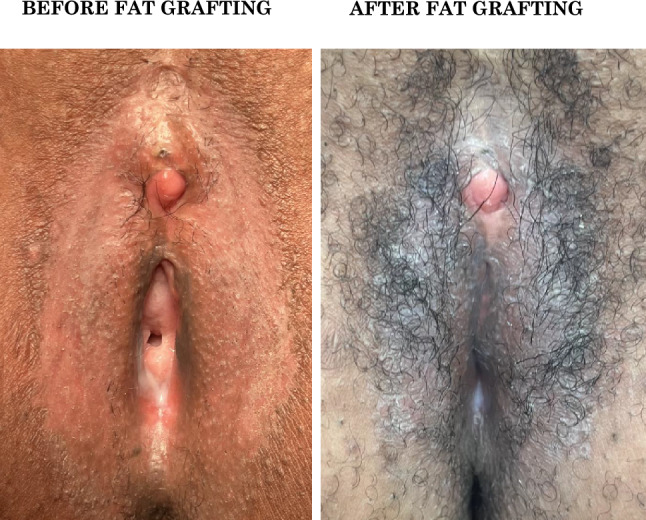
Representative clinical case 3: tissue preloading with fat to allow further surgical reconstruction. The patient underwent inner labia, clitoral hood, and clitoral glans resection, with suture of the outer labia including the clitoral hood. According to WHO classification this is Type 3; according to VASS the form is SEVERE in the clitoral area, outer labia and inner labia (complete loss of the anatomical structures). She then underwent defibulation and subsequent clitoral reconstruction. She presented with scar in the clitoral area, outer labia and inner labia (left). The clitoral glans was exposed and she complained oversensitivity of the neo-clitoris (left). She requested clitoral hood and inner labia reconstruction. However, given the scar tissue in the clitoral area and the lack of adequate soft tissue bulk to allow a local flap, this was not achievable. After one fat grafting procedure she presented with improved tissue quality and increased volumes, particularly in the clitoral region, which will allow a subsequent clitoral hood reconstruction. Additionally, she was satisfied with the increased volumes and better coverage of the vaginal opening, and decided not to have inner labia reconstruction

FG can also be applied as a preparatory step for more complex multistaged reconstructive procedures. For instance, FG to the inner labia can help correct scarring or enhance the subcutaneous tissue bulk, creating a better foundation for subsequent surgical intervention such as inner labia reconstruction. Similarly, FG in the clitoral area can be used to increase the subcutaneous tissue thickness of the clitoral hood when additional tissue is required for staged reconstructions, such as clitoral hood reconstruction, in cases where there is insufficient subcutaneous tissue to raise a local flap. By increasing the subcutaneous volumes with fat in the scarred retracted tissue, the overall soft tissue bulk is increased, and this can be critical for future procedures (Fig. 7). This approach, priorly described in the literature by our team as ‘tissue preloading with fat’ or ‘lipobed’, is often used in plastic surgery for irradiated tissues or sclerotic areas to improve tissue elasticity and subcutaneous tissue volumes, allowing for better surgical outcomes in later staged procedures [25, 40–43].

Another important consideration is that volumetric fat grafting is preferable over other surgical procedures, such as inner labia reconstruction, because it eliminates the risk of postoperative scarring. Inner labia reconstruction is a more complex procedure involving local flaps, which inevitably results in scar formation, and scar tissue may increase the risk of perineal tearing during a potential future vaginal childbirth. Women who have undergone FGM should not be regarded solely as "FGM cases"; their care must be managed holistically, taking into account factors such as lifestyle, age, and also, in younger patients, the potential for future pregnancy.

Therefore, the adaptability of FG to target specific areas of concern—whether it is to reduce scarring, improve volume, correct asymmetries, enhance tissue elasticity, or a combination of the above—makes it a highly versatile and promising intervention for FGM survivors.

Its ability to be combined with other reconstructive techniques further solidifies its role as a valuable resource in the health management of FGM women. Successful outcomes largely depend on careful patient selection and communication. Understanding each patient’s expectations and goals is crucial for providing a satisfactory and personalized approach.

### Strengths and Limitations

This initial pilot study represents the first report in the literature on the use of FG in FGM, and therefore marks the beginning of exploration of this treatment in FGM-related vulvar scarring, offering promising results, with a potential impact on millions of women worldwide. FG is minimally invasive and easily reproducible with limited costs, making it accessible even in low-resource settings and an attractive option for widespread implementation. Additionally, this treatment has proven safe, with only minor complications reported.

The study’s robustness is attributed to its use of validated PROMs along with a clinical scale evaluated by physicians, to assess outcomes both from the patients’ and doctors’ perspective. These tools add reliability to the assessments of patient satisfaction and procedural efficacy. This study, however, presents limitations. The small sample size diminishes the broader applicability of the results, and the limited postoperative follow-up fails to provide insight into the long-term durability of the effects observed. Furthermore, the evaluation of outcomes depended on subjective assessments; thus, future investigations should employ objective methods to measure tissue stiffness and histological alterations.

Although this research offers promising initial findings, it primarily acts as a foundation. Additional studies are essential before this technique can be implemented as a FGM treatment on a wider scale. There is an urgent need for clinical and histological investigations to validate the long-term effectiveness of the procedure in FGM scar.

## Conclusions

FGM constitutes a form of violence and human rights’ violation, offering no health benefits and causing harm [45]. The most impactful long-term consequence of FGM is the development of hypertrophic scars, which not only impair physical function but also lead to additional long-term health issues.

As a treatment for FGM-related scarring, FG offers a low-cost, minimally invasive solution with global applicability, particularly in low-resource settings. By targeting and reducing scar tissue, FG has the potential to reduce long-term morbidity associated with FGM, ultimately lowering the economic burden on public health systems globally.

Therefore, this minimally invasive intervention has far-reaching implications, providing a cost-effective and scalable solution to improve the overall well-being of millions of women living worldwide with a form of FGM.
